# Neuropathic Na_v_1.3-mediated sensitization to P2X activation is regulated by protein kinase C

**DOI:** 10.1186/1744-8069-7-14

**Published:** 2011-02-11

**Authors:** Gary Mo, Rebecca Grant, Dajan O'Donnell, David S Ragsdale, Chang-Qing Cao, Philippe Séguéla

**Affiliations:** 1 Montreal Neurological Institute and The Alan Edwards Centre for Research on Pain, Department of Neurology & Neurosurgery, McGill University, Montreal, Canada; 2 Department of Bioscience, AstraZeneca R&D Montreal, Montreal, Canada

## Abstract

**Background:**

Increased neuronal excitability and spontaneous firing are hallmark characteristics of injured sensory neurons. Changes in expression of various voltage-gated Na^+ ^channels (VGSCs) have been observed under neuropathic conditions and there is evidence for the involvement of protein kinase C (PKC) in sensory hyperexcitability. Here we demonstrate the contribution of PKC to P2X-evoked VGSC activation in dorsal root ganglion (DRG) neurons in neuropathic conditions.

**Results:**

Using the spinal nerve ligation (SNL) model of neuropathic pain and whole-cell patch clamp recordings of dissociated DRG neurons, we examined changes in excitability of sensory neurons after nerve injury and observed that P2X3 purinoceptor-mediated currents induced by α,β-meATP triggered activation of TTX-sensitive VGSCs in neuropathic nociceptors only. Treatment of neuropathic DRGs with the PKC blocker staurosporine or calphostin C decreased the α,β-meATP-induced Na^+ ^channels activity and reversed neuronal hypersensitivity. In current clamp mode, α,β-meATP was able to evoke action-potentials more frequently in neuropathic neurons than in controls. Pretreatment with calphostin C significantly decreased the proportion of sensitized neurons that generated action potentials in response to α,β-meATP. Recordings measuring VGSC activity in neuropathic neurons show significant change in amplitude and voltage dependence of sodium currents. In situ hybridization data indicate a dramatic increase in expression of embryonic Na_v_1.3 channels in neuropathic DRG neurons. In a CHO cell line stably expressing the Na_v_1.3 subunit, PKC inhibition caused both a significant shift in voltage-dependence of the channel in the depolarizing direction and a decrease in current amplitude.

**Conclusion:**

Neuropathic injury causes primary sensory neurons to become hyperexcitable to ATP-evoked P2X receptor-mediated depolarization, a phenotypic switch sensitive to PKC modulation and mediated by increased activity of TTX-sensitive VGSCs. Upregulation in VGSC activity after injury is likely mediated by increased expression of the Na_v_1.3 subunit, and the function of the Na_v_1.3 channel is regulated by PKC.

## Background

Neuronal hypersensitivity is a hallmark feature of neuropathic pain. The ATP-gated ionotropic P2X3 receptor [1,2] has been demonstrated to play significant roles in neuronal hyperexcitability and neuropathic pain in damaged sensory neurons [3-6]. To date, however, there is no clear understanding of how the P2X3 receptor influences neuronal hypersensitivity under neuropathic pain conditions. If the P2X3 receptor contributes directly to neuronal hypersensitivity, one might expect an upregulation of its function after nerve damage. Consistent with this hypothesis, some studies have described increased P2X3 expression following nerve injury [7,8]. However, others have reported evidence of decreased P2X3 expression in neuropathic conditions [9] with lowered or unchanged level of P2X receptor function in subsets of dorsal root ganglion (DRG) neurons [10]. Indeed, while ATP evokes strong responses from neuropathic sensory neurons, it is not clear whether this is a direct effect of increased P2X3 currents, or an indirect effect reflecting increased intrinsic neuronal excitability.

Numerous reports have demonstrated an association between neuropathic injuries and changes in the expression of voltage-gated sodium channels (VGSC) [11]. One of the most dramatic VGSC alterations after neuropathic injury is the upregulation of the embryonic Na_v_1.3 α subunit [12]. The Na_v_1.3 α subunit is highly expressed in embryonic DRG neurons, but is developmentally regulated such that it is only weakly expressed in adult DRG neurons [13]. Regulation of gene expression is not the only mechanism known to modulate VGSC function in neurons. Changes in phosphorylation states mediated by serine/threonine protein kinases have been shown to directly affect functional properties of Na_v_1.2 [14], Na_v_1.7 and Na_v_1.8 [15,16]. Involvement of serine/threonine protein kinases, especially protein kinase C (PKC), in neuropathic pain has been clearly demonstrated [17,18]. However, a modulatory role of PKC on the Na_v_1.3 subunit has yet to be determined.

The aim of the present study is to understand how P2X3 receptors and Na_v_1.3 VGSCs contribute to neuronal hyperexcitability in neuropathic DRG neurons. Here we report that upregulation of Na_v_1.3 increases intrinsic neuronal excitability, whereas ATP-gated currents through P2X3 receptors contribute sufficient depolarization to elicit spikes in damaged DRG neurons. We also show that PKC modulates neuronal hyperexcitability by regulating the function of Na_v_1.3. These data provide new insights into how injured peripheral nociceptors mediate abnormal ectopic firing that could sustain neuropathic pain.

## Materials and methods

### Neuropathic model

This study was conducted under a protocol that has been approved by an ethical committee. The animals were kept and experiments were performed at AstraZeneca R&D Montréal which has accreditation from CCAC (Canadian Council on Animal Care), AAALAC (Association for the Assessment and Accreditation of Laboratory Animal Care) and/or approved by AZ GVC (AstraZeneca Global Veterinary Council) for study conduct.

Spinal nerve ligations (SNL) at L5 and L6 levels were carried out in Sprague-Dawley male rats weighing 125-150 g following a protocol previously described by Kim and Chung [19]. In summary, after inducing deep anaesthesia using isoflurane inhalation, a 2 cm dorsal incision was made approximately from L3 and S2 vertebral regions. Paraspinal muscles on the left side were detached from the spinous processes in order to reveal the L5 and L6 spinal nerves. Once the nerves were cleared from connective tissue, a tight ligation was made on each nerve using a 4-0 silk thread. The muscles were then moved back to the appropriate regions along the vertebrae then the incision was sutured and antibiotic ointment was applied. During surgery the animals were given a constant flow of isoflurane inhalation to maintain deep anaesthesia.

### Neuronal and non-neuronal cell culture

DRGs from lumbar segments L4-L6 were extracted from naïve male Sprague-Dawley rats weighing 340-400 g (Charles River Canada) under deep anaesthesia induced by isoflurane.

For the SNL conditions, L5 and L6 lumbar segment DRGs on the ipsilateral side of the ligation were harvested at 35-40 days post-injury under deep anaesthesia induced by isoflurane.

Extracted DRGs were placed in ice-cold oxygenated DMEM (Gibco) for removal of connective tissue and dura mater. The isolated DRGs were then placed into DMEM containing 1 mg/mL of papain and 2 mg/mL collagenase type II (Sigma-Aldrich) and incubated for 1 h at 37°C and 100% humidity. After enzymatic digestion, the DRGs were transferred into DMEM containing 10% FBS and 1% L-glutamine, dissociated into single neurons by means of trituration using fire-polished pipettes. Dissociated neurons were then plated onto 35 mm cell culture dishes (Sarstedt) coated with laminin (BD Bioscience) and poly-D-lysine (Sigma-Aldrich), and cultured for 48 h at 37°C and 100% humidity in F-12 media (Gibco) containing 10% FBS, 1% L-glutamine as well as 100 U/mL penicillin and streptomycin.

CHO cells stably expressing human Na_v_1.3 α subunit (cell line CNahIII-12, [20]) were cultured in IMDM supplemented with 10% fetal bovine serum, 1% sodium hypoxanthine and thymidine (HT) supplement, 1% MEM non-essential amino acids, 100 U/mL Penicillin and streptomycin, and 400 μg/mL G418 (Gibco).

### Patch-clamp recordings in DRG neurons and CHO cells

Whole-cell patch-clamp recordings on DRG neurons were conducted using micropipettes produced by pulling borosilicate glass tubes using a P-97 puller (Sutter Instrument, Novato, CA), and fire-polishing with a MP-830 microforge (Narishige, Tokyo, Japan) to a tip resistance of 0.8-1 MΩ when filled with pipette solution, pH 7.2, containing (in mM): 140 CsCl, 10 NaCl, 1 EGTA and 5 HEPES. During recordings the neurons were constantly perfused with a solution at pH 7.4, comprised of (in mM): 98 choline-Cl, 42 NaCl, 3 KCl, 2 CaCl_2_, 2 MgCl_2_, 10 HEPES, 10 glucose, 25 TEA and 3 4-AP.

Membrane currents were recorded using an Axopatch 200B amplifier, digitized with a Digidata 3200A interface (Axon Instruments, Foster City, CA.), and acquired at a frequency of 5 kHz using pClamp 9. Voltage dependence of activation was determined from peak currents in response to 50 ms test pulses ranging from -80 to +90 mV.

Whole-cell patch-clamp recordings were also carried out in CHO cells stably expressing the Na_v_1.3 α subunit. The same voltage protocol was conducted using micropipettes with a tip resistance of 2-4 MΩ filled with a solution at pH 7.2, containing (in mM): 105 Cs-Asp, 10 CsCl, 10 NaCl, 5 EGTA and 10 HEPES. During recordings the cells were constantly perfused with an external solution at pH 7.4 containing (in mM): 130 NaCl, 4 KCl, 1.5 CaCl_2_, 1 MgCl_2_, 10 HEPES and 5 glucose. For CHO cell recordings, compensation for leak current was carried out using the P/4 protocol included in the pClamp 9 acquisition software. For all recordings the membrane capacitance (C_m_) and series resistance (R_s_) were measured through the peak amplitude and decay constant of transients induced by repetitive depolarizing pulses of 10 mV.

### Tissue collection and preparation

Adult male Sprague-Dawley rats (Charles River, Saint-Constant, Quebec, Canada) were euthanized by decapitation. Spinal cords with DRG attached were rapidly dissected, snap-frozen at -40°C in isopentane for 20 s and stored at -80°C. Frozen spinal cord tissue with DRGs attached were transversely cryosectioned at 14 μm and thaw-mounted onto Superfrost Plus slides (VWR, Montreal, Quebec, Canada). Slides were stored at -80°C until processing for *in situ *hybridization.

### Na_V_1.3 riboprobe design and synthesis

A 409-base pair fragment spanning nt. 6325-6733 in the 3'-untranslated region of the rat Na_v_1.3 gene (accession number NM_013119) was amplified in PCR from rat genomic DNA using 5'-TATCCGTGTCAACTGGAC-3' as the forward primer and 5'-ACTTGTGGACTTAGCAAC-3' as the reverse primer. PCR cycling (reaction mix: 1 ng of genomic DNA, 0.2 μM of each dNTP, 1 mM of each primer, 5 U of *Taq *DNA polymerase in 1 × *Taq *DNA polymerase buffer) was 3 min at 95°C, followed by 25 cycles of 30 s at 95°C, 45 s at 50°C and 30 s at 72°C. The amplicon was isolated on an agarose gel using the QIAquick gel extraction kit (Qiagen, Mississauga, Ontario, Canada) and was ligated into pGEM-T-Easy vector (Promega, Nepean, Ontario, Canada). Plasmid from a single clone was purified using HiSpeed Plasmid Midi kit (Qiagen). The Na_v_1.3 plasmid was linearized using NcoI and SpeI restriction enzymes (Promega). Antisense riboprobes were transcribed *in vitro *using SP6 RNA polymerase (Promega) and radiolabeled with ^35^S-UTP and ^35^S-CTP (800 Ci/mmol; Amersham Biosciences, Inc). Following transcription, the Na_v_1.3 DNA template was digested with DNAse I (Promega) and subsequently purified using G-50 Sepharose microspin columns (GE Healthcare). The quality of labeled riboprobes was verified by polyacrylamide-urea gel electrophoresis and scintillation counting.

### In situ hybridization (ISH) and histological analysis

ISH was performed on rat tissue sections as previously described (Ahmad et al 2007). Briefly, tissue sections were fixed with 4% paraformaldehyde, rinsed 3 times in 2 × standard sodium citrate buffer (2 × SSC), equilibrated in 0.1 M triethanolamine, and treated with 0.25% acetic anhydride in 0.1 M triethanolamine. After a rinse in 2 × SSC and dehydration through an ethanol series (50-100%), hybridization was performed in a buffer containing 75% formamide (Sigma), 600 mM NaCl, 10 mM Tris-HCl (pH 7.5), 1 mM EDTA, 1 × Denhardt's solution (Sigma), 50 μg/ml denatured salmon sperm DNA (Sigma), 10% dextran sulfate (Sigma), 20 mM dithiothreitol, and [^35^S]-labeled cRNA probe (20 × 106 cpm/ml) at 55°C overnight in chambers humidified with 75% formamide.

Following hybridization, slides were rinsed twice in 2 × SSC at room temperature, treated with 20 μg/ml RNase IA in RNase buffer (25 mM NaCl, 5 mM Tris-HCl pH 7.5, 0.5 mM EDTA pH 8.0) for 45 minutes at 37°C, and washed to a final stringency of 0.1 × SSC at 70°C. Sections were then dehydrated and exposed to Kodak Biomax MR-2 film. After exposed to film, the slides were dipped in Kodak NTB2 emulsion and exposed for 8 weeks at 4°C prior to development and counterstaining with hematoxylin and eosin.

Film autoradiograms were digitized with a high-resolution Xillix Microimager digital camera via the MCID image analysis system (Imaging Research, St-Catharines, Ontario, Canada). Bright- and darkfield photomicrographs of emulsion-dipped tissue sections were captured using a Leica (DMRBE/DM 4000B) microscope equipped with a Leica DFC490 digital camera. Digital images were transferred to Adobe Photoshop 7.0 for minimal image processing.

### Statistical analysis

Data are presented as mean ± S.E.M. All statistical analyses for the difference in means were carried out using unpaired as well as paired Student's *t *test and one-way ANOVA followed by Bonferroni's multiple comparison tests. Normalized data were analyzed using nonparametric Mann-Whitney U test.

## Results

### Neuropathic vs Naïve Purinergic Response

**Figure 1:**
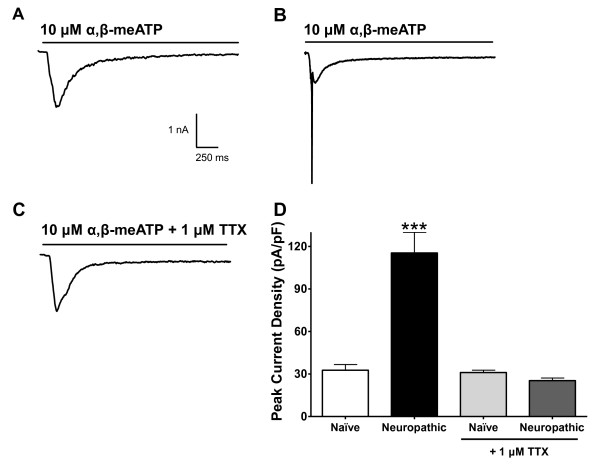
**Hyperexcitability of neuropathic DRG neurons to P2X activation and reversal by TTX**. Representative traces of P2X current responses induced by 10 μM α,β-meATP and recorded from cultured rat DRG neurons. (A) Recordings in control (naïve) neurons (B) in neuropathic SNL neurons, and (C) in neuropathic SNL neurons in the presence of 1 μM TTX. (D) Current densities of peak P2X responses from naïve and neuropathic DRG neurons, in absence and presence of 1 μM TTX (N = 6-17, *** *p *< 0.0005).

The overall morphology of DRG neurons was indistinguishable between naïve and neuropathic conditions. Three distinct populations of neurons (small, medium and large diameter) were present in all cultures. For the purpose of this study, recordings were conducted only on small diameter DRG neurons nociceptors, based on cell body diameter of <30 μm, with functional P2X3 receptors as determined by rapidly activating and fast desensitizing current kinetics in response to the selective agonist α,β-meATP. In DRG neurons from both control and neuropathic animals, application of 10 μM α,β-meATP resulted in inward current (Figure 1A, B); however, in the neuropathic DRGs, α,β-meATP elicited a large transient spike, which was not observed in control DRGs. We hypothesized that this large inward spike in neuropathic neurons might be caused by activation of VGSCs. In other words, in neuropathic conditions, a small initial depolarization caused by the inward current through P2X receptors and not completely compensated by the clamp might be sufficient to trigger a regenerative inward sodium spike. To test this hypothesis, we applied a saturating concentration of TTX (1 μM) and found that it selectively inhibited the inward spike, leaving the purinergic ionotropic response at levels similar to naïve neurons (Figure 1C, D). These data suggest that after neuropathic injury, small diameter DRG neurons express large TTX-sensitive voltage-dependent sodium currents. It should be noted that we cannot be certain that the recorded purinergic currents are purely mediated by homomeric P2X3 receptors since it has been reported that neuropathic DRG neurons show increased expression of P2X2/3 heteromeric receptors [10], thus we will simply describe purinergic currents as P2X responses.

**Figure 2:**
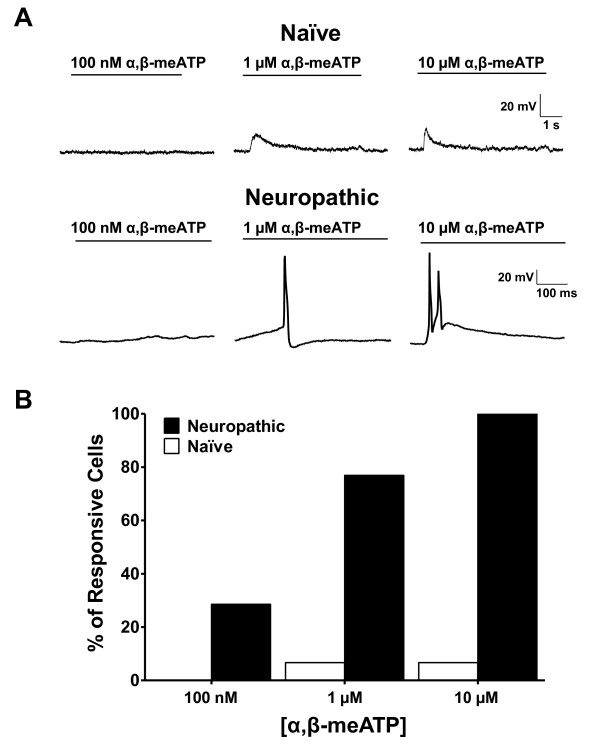
**P2X activation triggers action potentials in neuropathic DRG neurons**. (A) Representative traces obtained in current clamp recordings at resting membrane potential (no current injection), and action potentials induced by the application of increasing concentrations of α,β-meATP. (B) Percentage of naïve and neuropathic DRG neurons that generated action potentials in response to increasing concentrations of α,β-meATP. (N = 8-15, all cells were pooled).

To examine more directly how P2X receptor activation influences the electroresponsiveness of neuropathic DRG neurons, we conducted current-clamp recordings under resting conditions with no current injection, then stimulated the DRG neurons with an application of 100 nM, 1 μM or 10 μM α,β-meATP. In naïve DRG neurons, α,β-meATP elicited dose-dependent cellular depolarizations. However, these responses were generally not sufficient to trigger neuronal firing (Figure 2A). None of the naïve neurons tested at 100 nM α,β-meATP generated action potentials, and 1 μM or 10 μM α,β-meATP applications induced action potentials in about 7% of naïve DRG neurons. In contrast, a greater proportion of neuropathic DRG neurons fired action potentials in response to P2X stimulation. For example, 100 nM α,β-meATP induced neuronal firing in about 29% of DRG neurons, 1 μM α,β-meATP induced 77% of neurons to fire, and all neuropathic neurons generated action potentials in response to 10 μM α,β-meATP (Figure 2B; N = 8-15 for each concentration in each group).

### Hyperexcitability of Neuropathic DRGs is Sensitive to PKC

**Figure 3:**
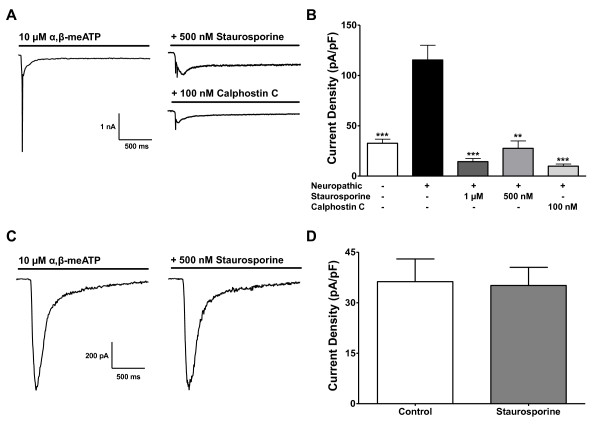
**Exaggerated α,β-meATP-evoked response after neuropathic injury is blunted by PKC inhibition**. (A) Sample traces of current responses to 10 μM α,β-meATP in presence of PKC inhibitors staurosporine (500 nM) and calphostin C (100 nM) in neuropathic DRG neurons under voltage-clamp settings. (B) Current density of peak responses to 10 μM α,β-meATP in presence of PKC inhibitors (N = 4-17, ** *p *< 0.005 *** *p *< 0.0005, compared to the neuropathic group). (C) Representative traces of 10 μM α,β-meATP-evoked P2X3 responses in naïve DRG neurons (left), and after pre-incubation with 500 nM staurosporine (right). (D) Current density of 10 μM α,β-meATP-evoked P2X responses with and without 500 nM staurosporine (N = 5, non significant difference).

To test the sensitivity of α,β-meATP-mediated current responses of both naïve and neuropathic DRG neurons to PKC modulation, we used the wide-spectrum protein kinase blocker staurosporine. In voltage clamp experiments, we found that the fast spike recorded in response to α,β-meATP in neuropathic neurons was inhibited by 500 nM staurosporine (Figure 3A). The current density of the response to 10 μM α,β-meATP was decreased by 76% (neuropathic: 115.41 ± 14.59 pA/pF; + staurosporine: 27.57 ± 7.42 pA/pF; N = 4-9; Figure 3A, B). Responses to α,β-meATP in naïve DRG neurons were not sensitive to staurosporine (Figure 3C, D), nor were the responses sensitive to pre-incubation with PKC activator PMA (data not shown). The α,β-meATP-evoked transient spike in neuropathic neurons was also significantly inhibited by calphostin C, a more selective inhibitor of PKC (Figure 3A, B; neuropathic: 115.14 ± 14.59 pA/pF; + calphostin C: 9.88 ± 2.07 pA/pF; N = 6-9).

**Figure 4:**
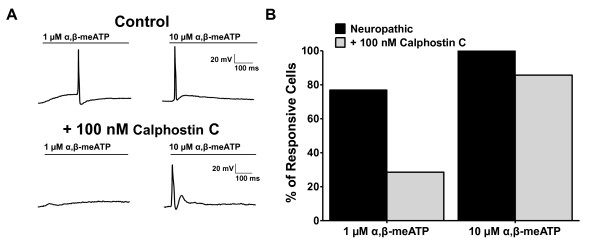
**Inhibition of α,β-meATP-evoked action potentials by the PKC blocker calphostin C in neuropathic DRG neurons**. (A) Typical recordings obtained from current-clamp experiments in neuropathic DRG neurons at resting membrane potential show action potentials generated in response to 1 and 10 μM α,β-meATP (upper panel), and their inhibition in the presence of 100 nM calphostin C (lower panel). (B) Percentage of DRG neurons that generated action potentials in response to α,β-meATP, in absence and presence of 100 nM calphostin C (N = 7-13).

To ascertain how PKC affected the electroresponsiveness of naïve versus neuropathic DRG neurons to P2X receptor activation, we conducted current-clamp experiments to measure the effects of inhibiting PKC with 100 nM calphostin C on action potentials induced by 1 and 10 μM α,β-meATP. The difference in electroresponsiveness between naïve and neuropathic neurons was less robust when we stimulated them with 100 nM α,β-meATP, thus it was not included in this set of experiments. In neuropathic DRG neurons activated with 1 μM α,β-meATP, only 29% of the neurons fired action potentials when pre-exposed to calphostin C, compared to 77% in absence of the PKC blocker (Figure 4A, B). When the neurons were activated with 10 μM α,β-meATP, 86% of neuropathic DRG neurons pre-treated with calphostin C generated action potentials, compared to 100% of untreated neuropathic neurons (Figure 4A, B; N = 7-13 for each concentration in each group).

### Neuropathic Injury-Induced Changes in Function and Expression of Voltage-Gated Sodium Channels

**Figure 5:**
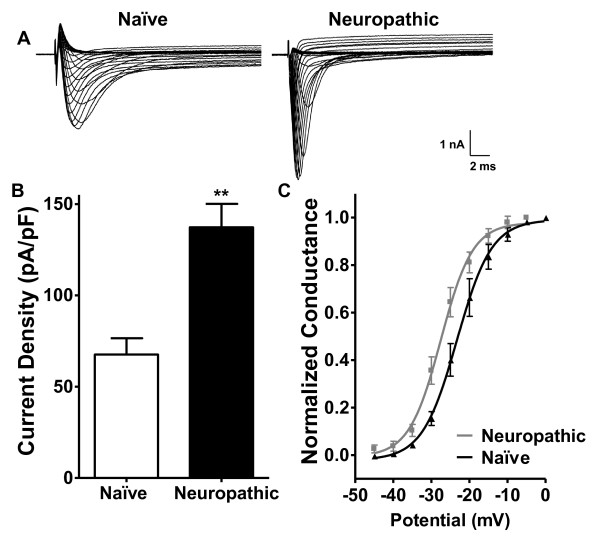
**Increased current amplitude and hyperpolarizing shift in voltage dependence of sensory sodium channels after neuropathic injury**. (A) Representative current-voltage relationship traces of VGSC currents recorded from naïve and neuropathic DRG neurons. (B) Analysis of peak current amplitudes of voltage-gated sodium currents recorded at V_max _in naïve and neuropathic conditions (N = 4-5 for both groups, ** *p *< 0.005). (C) Sodium channel conductance was fitted with the Boltzman function (see Materials and Methods) from voltage-gated currents recorded in naïve and neuropathic neurons (N = 4-5).

To investigate the role of VGSCs in the hyperexcitable responses of neuropathic DRG neurons to α,β-meATP-evoked depolarization, we examined voltage-activated sodium currents in voltage-clamp experiments. We observed dramatically larger sodium current amplitudes in neuropathic VGSCs, compared to naïve controls, over a range of test potentials (Figure 5A). At the peak of the current-voltage relationship, the current density measured in neuropathic DRG neurons was 200% larger than in naïve neurons (Figure 5B). We also observed a significant leftward shift (-5 mV) in the voltage dependence of activation in neuropathic neurons (Figure 5C). Therefore both an increased current amplitude and a negative shift in voltage dependence of activation of VGSCs contribute to the hypersensitivity of neuropathic DRG neurons to purinergic stimulation.

**Figure 6:**
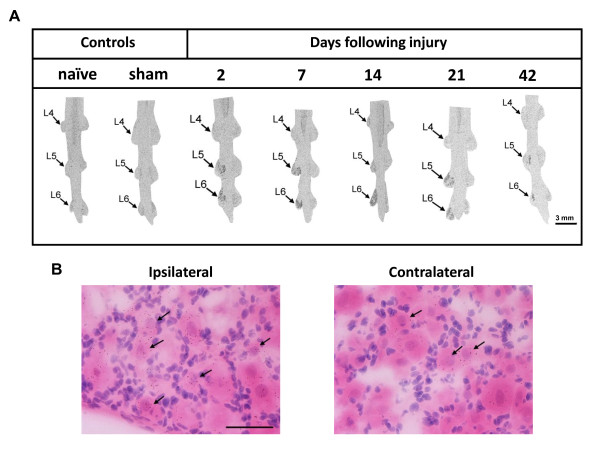
**Increased expression of Na_v_1.3 mRNA in DRG neurons following SNL injury**. (A) Autoradiographic in situ hybridization data showing increased levels of Na_v_1.3 mRNA in rat DRG neurons from 2 to 42 days after neuropathic injury. Arrows indicate the lumbar DRG level ipsilateral to the SNL injury. Note that the increase in Na_v_1.3 mRNA occurs in the ipsilateral (injured) L5 and L6 DRGs, but remains unchanged in (uninjured) L4 DRG and sham DRGs. Sham DRGs represent the 14 day post-injury time point. (B) High magnification (40×) images of emulsion-dipped sections showing increased Na_v_1.3 mRNA expression in cell bodies of small diameter DRG neurons (arrows) on the ipsilateral side of the nerve injury. Scale bar, 50 μm.

To investigate expression changes in VGSC, we performed in situ hybridization (ISH) experiments to examine alterations of mRNA levels in DRG tissue following SNL injury. We probed the expression of the Na_v_1.3 gene based on reports that this VGSC subunit increases significantly after SNL injury (see discussion) and axotomy [12]. Na_v_1.3 mRNA expression was qualitatively assessed in DRG sections from naïve, sham-operated and SNL-injured adult rats. In both naïve and sham-operated rats, very weak Na_v_1.3 mRNA expression was detected over a subset of small-, medium- and large-diameter neurons as identified using light microscopy (data not shown). In contrast, unilateral ligation of L5 and L6 spinal nerves resulted in robust upregulation of Na_v_1.3 mRNA expression within the corresponding ipsilateral DRGs (Figure 6A, B). The increased level of Na_v_1.3 mRNA expression was detected as early as 2 days post-injury, and remained upregulated at all time points examined in small-, medium- and large-diameter neurons. No changes were detected in the intact L4 DRG, or in contralateral L5 and L6 DRGs from SNL injured rats.

### PKC Modulation of Na_v_1.3 Currents

**Figure 7:**
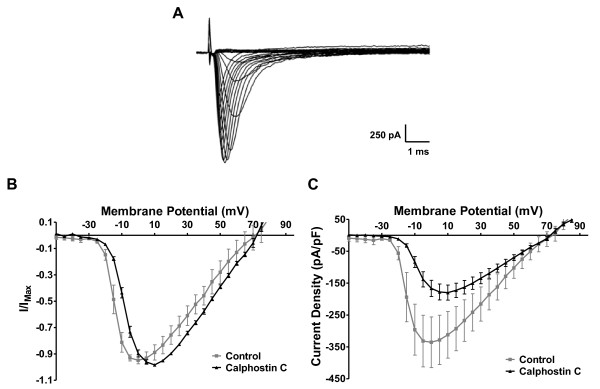
**Na_v_1.3 activity is positively regulated by PKC**. (A) Membrane potential depolarization induced Na_v_1.3-mediated sodium currents in Na_v_1.3-transfected CHO cells evoked using stimulus protocol described in methods section. (B) Current-voltage relationship of Na_v_1.3 activity in the presence and absence of 100 nM calphostin C (N = 6-8). (C) Voltage-dependent current density mediated by Na_v_1.3 channels in CHO cells in the presence and absence of 100 nM calphostin C (N = 6-8).

As we did not observe any modulatory role of PKC on the function of endogenous P2X3 receptors in DRG neurons (Figure 3C, D), it is unlikely that PKC is modulating neuronal excitability by altering P2X3 function. A more likely mechanism for the effects of PKC on the excitability of neuropathic DRG neurons is a modulation of the activity of Na_v_1.3 channels. Because native DRG neurons express a wide range of VGSC subtypes, and since no pharmacological tools are available to isolate the Na_v_1.3 activity, we tested this hypothesis in voltage-clamp experiments on a CHO cell line stably expressing the Na_v_1.3 subunit [20]. These cells displayed robust and reproducible voltage-gated sodium currents (Figure 7A). After treatment with the PKC blocker calphostin C, the voltage-dependence of Na_v_1.3 activity was shifted by 10 mV in the depolarizing direction (Figure 7B). PKC inhibition also significantly reduced the amplitude of Na_v_1.3 currents (Figure 7C). The current density at peak activation was reduced by 46% by calphostin C (control: 335.46 ± 79.41 pA/pF; + calphostin C: 179.70 ± 23.53 pA/pF; N = 6-8).

## Discussion

The present study suggests that activation of P2X receptors elicits excessive firing in neuropathic DRG neurons indirectly, through activation of upregulated Na_v_1.3 sodium channels. We also show that VGSC-mediated hyperexcitability is modulated by PKC.

### Activation of P2X Receptors Elicits Ectopic Action Potentials

Neuropathic injury models such as SNL induce a wide array of changes in the physiology of DRG neurons. Although SNL neurons display excessive firing of action potentials in response to P2X activation, we cannot conclude that the ability to trigger hypersensitive responses is exclusive to P2X receptors. Indeed, we also observed similar abnormal action potential firing in a small number of neuropathic cells stimulated with the TRPV1 agonist capsaicin (data not shown). We did not observe any significant changes in the current amplitude of P2X receptors in neuropathic DRG neurons treated with a saturating concentration of TTX. This is in accordance to another study of P2X receptor activity in injured sensory neurons, where it was reported that the amplitude of α,β-meATP-evoked currents in neuropathic small diameter DRG neurons did not significantly differ from those recorded from naïve neurons [10]. These observations are consistent with the idea that neuropathic neurons are generally hyper-responsive to depolarizing stimuli because of their intrinsic, voltage-dependent hyperexcitability. P2X3 receptors are fast-desensitizing while other receptor-channels such as TRPV1 have slower kinetics of activation and desensitization [21]. Thus, ligand-gated cation channels with different kinetics may elicit specific patterns and/or durations of ectopic action potential firing.

### PKC Modulation of Neuronal Hyperexcitability

The involvement of PKC in neuropathic pain has been well demonstrated [22], and certain isoforms of PKC are high value targets for pain therapeutics [23]. However, the mechanism by which PKC regulates neuronal excitability remains elusive. The function of several sensory receptor-channels has been shown to be modulated by PKC, such as TRPV1 [24], P2X2/3 [25], and ASICs [26]. In agreement with reports demonstrating the role of protein kinases in neuropathic pain mechanisms [17,27,28], we show that sensitization to ATP of neuropathic neurons can be inhibited by the wide-spectrum protein kinase blocker staurosporine, as well as calphostin C, a more selective PKC inhibitor [29] shown to be effective against PKC isoforms involved in neuropathic pain [30].

We did not observe any effect of staurosporine or the PKC activator PMA on P2X3 responses to α,β-meATP in either naïve DRG neurons or HEK293 cells expressing homomeric P2X3 receptors (data not shown), which strongly suggests that PKC is not contributing to hyperexcitability through a direct modulation of P2X receptors in neuropathic DRG neurons. Our voltage-clamp recordings conducted in the presence of PKC inhibitors showed a decrease in the fast spike component of the inward current, corresponding to a reduction in voltage-gated sodium current, while the remaining P2X component was relatively unchanged. The modulation of sodium channel activity by calphostin C was also observed in our current-clamp experiments where we demonstrated that PKC inhibition attenuates the generation of action potentials in neuropathic neurons. In line with evidence demonstrating modulation of VGSCs by PKC [14-16,31-33], our results point to a major role for PKC-modulated VGSCs in the hypersensitivity of neuropathic DRG neurons to purinergic stimulation.

### Upregulation of Na_v_1.3 Sodium Channels Expression and Activity

In order to verify that PKC is regulating hyperexcitability through modulation of VGSC, we first needed to identify which VGSC α subunit is up-regulated in neuropathic DRG neurons. From our characterization of the changes to the VGSC profile following SNL, the increase in current amplitude and the hyperpolarizing shift in voltage-dependence observed in neuropathic neurons are likely caused by increased expression of Na_v_1.3. This is consistent with reports showing an increase in the expression of embryonic Na_v_1.3 α subunit expression in DRG neurons after axotomy [12] and SNL injury [34,35]. We cannot exclude possible changes in the function of other VGSC subunits, such as Na_v_1.7 and Na_v_1.8, which may contribute to neuropathic hyperexcitability; however, their expression levels have been shown to decrease after neuropathic injury [36,37]. Under normal conditions, Na_v_1.8 is the main generator of action potentials in small diameter DRG neurons. With respect to activation, Na_v_1.8 has a V_0.5 _of -16 to -21 mV, whereas for Na_v_1.3 the V_0.5 _is -23 mV [38]. The difference in voltage-dependence of the two Na_v _subunits corresponds to the difference in voltage-dependence that we observed between neuropathic and naïve DRG neurons, suggesting that Na_v_1.3 is a major contributor to the VGSC-mediated currents in neuropathic neurons.

Increased expression of the Na_v_1.3 α subunit following SNL injury was confirmed through our ISH data. The increased expression level was confined to the injured L5 and L6 DRGs and remained unaltered on the contralateral (uninjured) DRGs. Changes in expression of VGSC subunit mRNA have been reported in both rodents and humans [12,34,37]. An increase in expression of Na_v_1.3 has been observed in trigeminal neuralgia patients [37], suggesting a role for Na_v_1.3 modulation in pathological pain. Neuronal VGSCs are heteromeric complexes containing α subunits associated with β subunits. These auxiliary subunits have been shown to modulate the function of various α subunits [39]. Of the three β subunits identified so far, the most relevant here is the β3 subunit because of its co-localization with Na_v_1.3 in small diameter DRG neurons. Furthermore, the expression of both proteins increases after neuropathic injury within the same population of neurons [12,40]. However, the modulatory effects of the β3 subunit on the Na_v_1.3 subunit are not clear. Some have reported a depolarizing shift in the voltage-dependence of activation and inactivation of Na_v_1.3 [41,42], others reported no change in voltage-dependence of activation, but did observe a decrease in the rate of recovery from inactivation [20]. Unlike other α subunits, the current density of Na_v_1.3 does not seem to be affected by the presence of β subunits [20,40]. In any case, the potential modulation of Na_v_1.3 by the auxiliary β3 subunit does not fit with the changes in the function of Na_v_1.3 after neuropathic injury that we are reporting here. However, because all these studies were based on the use of transfected cell lines, the exact role of native β3 subunits in sensory neurons is still unknown so they could possibly facilitate neuronal hyperexcitability. While some observed that treatment with antisense oligodeoxynucleotides targeting Na_v_1.3 reversed changes in pain responses associated with nerve injury [43,44], others reported that decreasing expression of Na_v_1.3 was not sufficient to attenuate neuropathic pain behaviours [45,46]. There is currently no consensus on the involvement of Na_v_1.3 function in mediating neuropathic pain. Therefore, until a selective blocker is available for clinical evaluation, its contribution to pain pathology will remain unclear.

### PKC Regulates Na_v_1.3 Activity

After identifying Na_v_1.3 as the VGSC α subunit predominantly responsible for the hyperexcitability following neuropathic injury, we examined the PKC modulation of its function isolated in a heterologous system. From currents recorded from CHO- Na_v_1.3 cells, it was clear that PKC enhances the function of Na_v_1.3 by increasing its activity at more hyperpolarizing potentials and by increasing its current amplitude, a combination that could dramatically boost the sensitivity of DRG nociceptors to a range of depolarizing stimuli. Several VGSC isoforms have been shown to be modulated by PKC [15,16,32], but we report here for the first time the positive regulation of Na_v_1.3 function by PKC in native as well as recombinant cell preparations. The molecular mechanisms of this modulation require further investigation to know if Na_v_1.3 is directly phosphorylated by PKC such as the Na_v_1.8 subunit [47]. In any case, the identification of phosphorylated domain(s) in the Na_v_1.3 subunit or associated proteins could provide valuable information for the design of novel neuropathic pain therapeutics.

## Conclusions

The present study shows that injury-induced hyper-responsiveness of DRG neurons to P2X activation is mediated by increased expression of Na_v_1.3 sodium channel subunit, and not due to any significant change in the function of P2X receptors. We also provide evidence for the first time that Na_v_1.3-mediated hyperexcitability can be modulated by PKC, which directly regulates the activity of Na_v_1.3 channels. This study emphasizes the importance of VGSCs in injury-induced increases in excitability to algogenic mediators, and provides new insights into functional regulation of Na_v_1.3 in injured peripheral neurons.

## Competing interests

The authors declare that they have no competing interests.

## Authors' contributions

GM and RG designed and performed the experiments. GM, RG, DO, DSR, CQC and PS participated in the manuscript writing. DO, DSR, CQC and PS provided guidance on experimental design and data interpretation, CQC and PS supervised the progress of the study. All the authors have read and approved the final manuscript.

## References

[B1] Dowd E McQueen DS Chessell IP Humphrey PP P2X receptor-mediated excitation of nociceptive afferents in the normal and arthritic rat knee joint Br J Pharmacol 1998 125 341 346 10.1038/sj.bjp.0702080 9786507 PMC1565628

[B2] Rong W Burnstock G Spyer KM P2X purinoceptor-mediated excitation of trigeminal lingual nerve terminals in an in vitro intra-arterially perfused rat tongue preparation J Physiol 2000 524 Pt 3 891 902 10.1111/j.1469-7793.2000.00891.x 10790166 PMC2269894

[B3] Barclay J Patel S Dorn G Wotherspoon G Moffatt S Eunson L Abdel'al S Natt F Hall J Winter J Functional downregulation of P2X3 receptor subunit in rat sensory neurons reveals a significant role in chronic neuropathic and inflammatory pain J Neurosci 2002 22 8139 8147 12223568 10.1523/JNEUROSCI.22-18-08139.2002 PMC6758070

[B4] Burnstock G Wood JN Purinergic receptors: their role in nociception and primary afferent neurotransmission Curr Opin Neurobiol 1996 6 526 532 10.1016/S0959-4388(96)80060-2 8794102

[B5] Cockayne DA Hamilton SG Zhu QM Dunn PM Zhong Y Novakovic S Malmberg AB Cain G Berson A Kassotakis L Urinary bladder hyporeflexia and reduced pain-related behaviour in P2X3-deficient mice Nature 2000 407 1011 1015 10.1038/35039519 11069181

[B6] North RA Verkhratsky A Purinergic transmission in the central nervous system Pflugers Arch 2006 452 479 485 10.1007/s00424-006-0060-y 16688467

[B7] Novakovic SD Kassotakis LC Oglesby IB Smith JA Eglen RM Ford AP Hunter JC Immunocytochemical localization of P2X3 purinoceptors in sensory neurons in naive rats and following neuropathic injury Pain 1999 80 273 282 10.1016/S0304-3959(98)00225-5 10204740

[B8] Xiang Z Xiong Y Yan N Li X Mao Y Ni X He C LaMotte RH Burnstock G Sun J Functional up-regulation of P2X 3 receptors in the chronically compressed dorsal root ganglion Pain 2008 140 23 34 10.1016/j.pain.2008.07.006 18715715 PMC2667225

[B9] Bradbury EJ Burnstock G McMahon SB The expression of P2X3 purinoreceptors in sensory neurons: effects of axotomy and glial-derived neurotrophic factor Mol Cell Neurosci 1998 12 256 268 10.1006/mcne.1998.0719 9828090

[B10] Kage K Niforatos W Zhu CZ Lynch KJ Honore P Jarvis MF Alteration of dorsal root ganglion P2X3 receptor expression and function following spinal nerve ligation in the rat Exp Brain Res 2002 147 511 519 10.1007/s00221-002-1263-x 12444483

[B11] Devor M Sodium channels and mechanisms of neuropathic pain J Pain 2006 7 S3 S12 10.1016/j.jpain.2005.09.006 16426998

[B12] Waxman SG Kocsis JD Black JA Type III sodium channel mRNA is expressed in embryonic but not adult spinal sensory neurons, and is reexpressed following axotomy J Neurophysiol 1994 72 466 470 7965028 10.1152/jn.1994.72.1.466 PMC2605356

[B13] Felts PA Yokoyama S Dib-Hajj S Black JA Waxman SG Sodium channel alpha-subunit mRNAs I, II, III, NaG, Na6 and hNE (PN1): different expression patterns in developing rat nervous system Brain Res Mol Brain Res 1997 45 71 82 10.1016/S0169-328X(96)00241-0 9105672

[B14] Chen Y Yu FH Surmeier DJ Scheuer T Catterall WA Neuromodulation of Na+ channel slow inactivation via cAMP-dependent protein kinase and protein kinase C Neuron 2006 49 409 420 10.1016/j.neuron.2006.01.009 16446144

[B15] Baker MD Protein kinase C mediates up-regulation of tetrodotoxin-resistant, persistent Na+ current in rat and mouse sensory neurones J Physiol 2005 567 851 867 10.1113/jphysiol.2005.089771 16002450 PMC1474230

[B16] Vijayaragavan K Boutjdir M Chahine M Modulation of Na_v_1.7 and Na_v_1.8 peripheral nerve sodium channels by protein kinase A and protein kinase C J Neurophysiol 2004 91 1556 1569 10.1152/jn.00676.2003 14657190

[B17] Cesare P Dekker LV Sardini A Parker PJ McNaughton PA Specific involvement of PKC-epsilon in sensitization of the neuronal response to painful heat Neuron 1999 23 617 624 10.1016/S0896-6273(00)80813-2 10433272

[B18] Malmberg AB Chen C Tonegawa S Basbaum AI Preserved acute pain and reduced neuropathic pain in mice lacking PKCgamma Science 1997 278 279 283 10.1126/science.278.5336.279 9323205

[B19] Kim SH Chung JM An experimental model for peripheral neuropathy produced by segmental spinal nerve ligation in the rat Pain 1992 50 355 363 10.1016/0304-3959(92)90041-9 1333581

[B20] Meadows LS Chen YH Powell AJ Clare JJ Ragsdale DS Functional modulation of human brain Na_v_1.3 sodium channels, expressed in mammalian cells, by auxiliary beta 1, beta 2 and beta 3 subunits Neuroscience 2002 114 745 753 10.1016/S0306-4522(02)00242-7 12220575

[B21] Caterina MJ Schumacher MA Tominaga M Rosen TA Levine JD Julius D The capsaicin receptor: a heat-activated ion channel in the pain pathway Nature 1997 389 816 824 10.1038/39807 9349813

[B22] Velazquez KT Mohammad H Sweitzer SM Protein kinase C in pain: involvement of multiple isoforms Pharmacol Res 2007 55 578 589 10.1016/j.phrs.2007.04.006 17548207 PMC2140050

[B23] Yonezawa T Kurata R Kimura M Inoko H PKC delta and epsilon in drug targeting and therapeutics Recent Pat DNA Gene Seq 2009 3 96 101 10.2174/187221509788654205 19519579

[B24] Plant TD Zollner C Kepura F Mousa SS Eichhorst J Schaefer M Furkert J Stein C Oksche A Endothelin potentiates TRPV1 via ETA receptor-mediated activation of protein kinase C Mol Pain 2007 3 35 10.1186/1744-8069-3-35 18001466 PMC2206006

[B25] Maruo K Yamamoto H Yamamoto S Nagata T Fujikawa H Kanno T Yaguchi T Maruo S Yoshiya S Nishizaki T Modulation of P2X receptors via adrenergic pathways in rat dorsal root ganglion neurons after sciatic nerve injury Pain 2006 120 106 112 10.1016/j.pain.2005.10.016 16360272

[B26] Baron A Deval E Salinas M Lingueglia E Voilley N Lazdunski M Protein kinase C stimulates the acid-sensing ion channel ASIC2a via the PDZ domain-containing protein PICK1 J Biol Chem 2002 277 50463 50468 10.1074/jbc.M208848200 12399460

[B27] Dina OA Barletta J Chen X Mutero A Martin A Messing RO Levine JD Key role for the epsilon isoform of protein kinase C in painful alcoholic neuropathy in the rat J Neurosci 2000 20 8614 8619 11069970 10.1523/JNEUROSCI.20-22-08614.2000 PMC6773162

[B28] Hua XY Chen P Yaksh TL Inhibition of spinal protein kinase C reduces nerve injury-induced tactile allodynia in neuropathic rats Neurosci Lett 1999 276 99 102 10.1016/S0304-3940(99)00818-6 10624801

[B29] Kobayashi E Nakano H Morimoto M Tamaoki T Calphostin C (UCN-1028C), a novel microbial compound, is a highly potent and specific inhibitor of protein kinase C Biochem Biophys Res Commun 1989 159 548 553 10.1016/0006-291X(89)90028-4 2467670

[B30] Norcini M Vivoli E Galeotti N Bianchi E Bartolini A Ghelardini C Supraspinal role of protein kinase C in oxaliplatin-induced neuropathy in rat Pain 2009 146 141 147 10.1016/j.pain.2009.07.017 19683395

[B31] Curia G Aracri P Colombo E Scalmani P Mantegazza M Avanzini G Franceschetti S Phosphorylation of sodium channels mediated by protein kinase-C modulates inhibition by topiramate of tetrodotoxin-sensitive transient sodium current Br J Pharmacol 2007 150 792 797 10.1038/sj.bjp.0707144 17279091 PMC2013870

[B32] Gold MS Levine JD Correa AM Modulation of TTX-R INa by PKC and PKA and their role in PGE2-induced sensitization of rat sensory neurons in vitro J Neurosci 1998 18 10345 10355 9852572 10.1523/JNEUROSCI.18-24-10345.1998 PMC6793376

[B33] Wada A Yanagita T Yokoo H Kobayashi H Regulation of cell surface expression of voltage-dependent Na_v_1.7 sodium channels: mRNA stability and posttranscriptional control in adrenal chromaffin cells Front Biosci 2004 9 1954 1966 10.2741/1314 14977601

[B34] Fukuoka T Kobayashi K Yamanaka H Obata K Dai Y Noguchi K Comparative study of the distribution of the alpha-subunits of voltage-gated sodium channels in normal and axotomized rat dorsal root ganglion neurons J Comp Neurol 2008 510 188 206 10.1002/cne.21786 18615542

[B35] Kim CH Oh Y Chung JM Chung K The changes in expression of three subtypes of TTX sensitive sodium channels in sensory neurons after spinal nerve ligation Brain Res Mol Brain Res 2001 95 153 161 10.1016/S0169-328X(01)00226-1 11687287

[B36] Boucher TJ Okuse K Bennett DL Munson JB Wood JN McMahon SB Potent analgesic effects of GDNF in neuropathic pain states Science 2000 290 124 127 10.1126/science.290.5489.124 11021795

[B37] Siqueira SR Alves B Malpartida HM Teixeira MJ Siqueira JT Abnormal expression of voltage-gated sodium channels Na_v_1.7, Na_v_1.3 and Na_v_1.8 in trigeminal neuralgia Neuroscience 2009 164 573 577 10.1016/j.neuroscience.2009.08.037 19699781

[B38] Catterall WA Goldin AL Waxman SG International Union of Pharmacology. XLVII. Nomenclature and structure-function relationships of voltage-gated sodium channels Pharmacol Rev 2005 57 397 409 10.1124/pr.57.4.4 16382098

[B39] Isom LL De Jongh KS Patton DE Reber BF Offord J Charbonneau H Walsh K Goldin AL Catterall WA Primary structure and functional expression of the beta 1 subunit of the rat brain sodium channel Science 1992 256 839 842 10.1126/science.1375395 1375395

[B40] Shah BS Stevens EB Gonzalez MI Bramwell S Pinnock RD Lee K Dixon AK beta3, a novel auxiliary subunit for the voltage-gated sodium channel, is expressed preferentially in sensory neurons and is upregulated in the chronic constriction injury model of neuropathic pain Eur J Neurosci 2000 12 3985 3990 10.1046/j.1460-9568.2000.00294.x 11069594

[B41] Cummins TR Aglieco F Renganathan M Herzog RI Dib-Hajj SD Waxman SG Na_v_1.3 sodium channels: rapid repriming and slow closed-state inactivation display quantitative differences after expression in a mammalian cell line and in spinal sensory neurons J Neurosci 2001 21 5952 5961 11487618 10.1523/JNEUROSCI.21-16-05952.2001 PMC6763143

[B42] Cusdin FS Nietlispach D Maman J Dale TJ Powell AJ Clare JJ Jackson AP The sodium channel {beta}3-subunit induces multiphasic gating in Na_V_1.3 and affects fast inactivation via distinct intracellular regions J Biol Chem 2010 285 33404 33412 10.1074/jbc.M110.114058 20675377 PMC2963402

[B43] Hains BC Klein JP Saab CY Craner MJ Black JA Waxman SG Upregulation of sodium channel Na_v_1.3 and functional involvement in neuronal hyperexcitability associated with central neuropathic pain after spinal cord injury J Neurosci 2003 23 8881 8892 14523090 10.1523/JNEUROSCI.23-26-08881.2003 PMC6740400

[B44] Hains BC Saab CY Klein JP Craner MJ Waxman SG Altered sodium channel expression in second-order spinal sensory neurons contributes to pain after peripheral nerve injury J Neurosci 2004 24 4832 4839 10.1523/JNEUROSCI.0300-04.2004 15152043 PMC6729453

[B45] Lindia JA Kohler MG Martin WJ Abbadie C Relationship between sodium channel Na_v_1.3 expression and neuropathic pain behavior in rats Pain 2005 117 145 153 10.1016/j.pain.2005.05.027 16061326

[B46] Nassar MA Baker MD Levato A Ingram R Mallucci G McMahon SB Wood JN Nerve injury induces robust allodynia and ectopic discharges in Na_v_1.3 null mutant mice Mol Pain 2006 2 33 10.1186/1744-8069-2-33 17052333 PMC1630424

[B47] Hudmon A Choi JS Tyrrell L Black JA Rush AM Waxman SG Dib-Hajj SD Phosphorylation of sodium channel Na_v_1.8 by p38 mitogen-activated protein kinase increases current density in dorsal root ganglion neurons J Neurosci 2008 28 3190 3201 10.1523/JNEUROSCI.4403-07.2008 18354022 PMC6670703

